# Association between hip muscle cross-sectional area and hip pain and function in individuals with mild-to-moderate hip osteoarthritis: a cross-sectional study

**DOI:** 10.1186/s12891-020-03348-5

**Published:** 2020-05-21

**Authors:** Waruna L. Peiris, Flavia M. Cicuttini, Maria Constantinou, Abbas Yaqobi, Sultana Monira Hussain, Anita E. Wluka, Donna Urquhart, Rod Barrett, Ben Kennedy, Yuanyuan Wang

**Affiliations:** 1 School of Public Health and Preventive Medicine, Monash University, 553 St Kilda Rd, Melbourne, VIC 3004 Australia; 2 School of Allied Health, Faculty of Health Sciences, Australian Catholic University, Banyo, Brisbane, Queensland 4014 Australia; 3 School of Allied Health Sciences and Menzies Health Institute Queensland, Griffith University, Gold Coast Campus, Gold Coast, Queensland 4222 Australia; 4 Qscan Radiology Clinics, Brisbane, Queensland Australia

## Abstract

**Background:**

To examine the associations between hip muscle cross-sectional area and hip pain and function in community-based individuals with mild-to-moderate hip osteoarthritis.

**Methods:**

This study included 27 participants with mild-to-moderate hip osteoarthritis. Cross-sectional area of hip muscles, including psoas major, rectus femoris, gluteus maximus, gluteus medius and minimus, adductor longus and magnus, obturator internus, and obturator externus, were measured from magnetic resonance images. Hip pain and function were evaluated using the Hip Disability and Osteoarthritis Outcome Score (HOOS) categorised into 5 subscales: pain, symptoms, activity of daily living, sport and recreation function, and hip-related quality of life (for each subscale 0 representing extreme problems and 100 representing no problems).

**Results:**

Mean age of the 27 participants was 63.2 (SD 7.6) years and 66.7% (*n* = 18) were female. After adjusting for age and gender, greater cross-sectional area of adductor longus and magnus was associated with a higher HOOS score in quality of life (regression coefficient 1.4, 95% confidence interval (CI) 0.2–2.7, *p* = 0.02), activity of daily living (regression coefficient 1.3, 95% CI 0.1–2.6, *p* = 0.04) and sport and recreation function (regression coefficient 1.6, 95% CI 0.1–3.0, *p* = 0.04). There was a trend towards an association between greater cross-sectional area of psoas major and a higher quality of life score (regression coefficient 3.6, 95% CI − 0.5 to 7.7, *p* = 0.08). The cross-sectional area of hip muscles was not significantly associated with HOOS pain or symptom score.

**Conclusion:**

Greater cross-sectional area of hip adductors was associated with better function and quality of life in individuals with mild-to-moderate hip osteoarthritis. Greater cross-sectional area of hip flexors might be associated with better quality of life. These findings, while need to be confirmed in longitudinal studies, suggest that targeting the hip adductor and flexor muscles may improve function and quality of life in those with mild-to-moderate hip osteoarthritis.

## Background

Osteoarthritis (OA) is one of the leading causes of disability worldwide, with disability-adjusted life years predicted to rise with the increasing age and prevalence of obesity in the population [1]. Hip OA has a life time prevalence of one in four people [2] and can be both painful and disabling, severely impacting the quality of life of an individual [3]. Current efforts to reduce the burden of hip OA include treatment for alleviation of pain and improvement of function that include exercise, weight reduction, acetaminophen, non-steroidal anti-inflammatory drugs, and intra-articular injections of corticosteroids and hyaluronates [4], with end-stage disease treated with total hip replacement.

Hip muscles are critical for movement of the trunk and legs and redistribution of segmental power when walking, and may also be important for stabilizing the hip joint [5, 6]. The force generated by a muscle is largely a function of the muscle’s physiological cross-sectional area (CSA) and the level of motor unit pool activation [7, 8]. There is evidence for generalized muscle weakness of the affected leg in people with unilateral hip OA, due to multifactorial mechanisms including a combination of reduced muscle size (atrophy), muscle inhibition, and decreased muscle quality [9]. More specifically, there is evidence of deficits in lower limb muscle strength and size in people with mild-to-moderate hip OA relative to healthy controls [10, 11], and biomechanical studies reported functional deficits during walking and sit-to-stand in participants with mild-to-moderate hip OA compared with healthy controls [12, 13] and altered muscle activity during gait in people with hip OA compared with healthy controls [14]. However, there are limited data examining the associations between hip muscle properties and patient-reported outcomes in pain and function. Previous studies have shown some evidence for an association between decreased hip adductor strength with groin pain [15, 16], and for a negative association between fiber CSA of gluteus medius muscle and hip pain [17]. These studies have only looked at a single muscle or limited groups of muscles, and investigations of a wider range of hip muscles are needed to establish a better understanding of the relationship between muscle weakness and functional outcomes in hip OA [9].

There is a need for studies to comprehensively examine hip muscles of different functional groups as possible modifiable factors for improving the management of hip OA at an early stage of the disease, where the opportunity to alter patient outcomes remains. The aim of this study was therefore to examine the associations between CSA of hip muscles of different functional groups and patient-reported hip pain and function in individuals with mild-to-moderate hip OA.

## Methods

### Participants

Individuals over the age of 45 years were recruited between June 2011 and October 2014 through advertisements, word of mouth, and hospital orthopaedic waiting lists. Volunteers were screened using the Harris Hip Score (HHS) [18] and radiographic examination. From 420 individuals who volunteered, 60 individuals with hip pain were eligible to complete the HHS and subsequently undergo radiographic screening evaluation for possible participation in the study. All participants were of Caucasian background. Weight-bearing anterior-posterior radiographs of the pelvis and hips were performed with feet internally rotated by 15 ± 5 degrees [19]. An experienced radiologist electronically scored radiographs for both hips based on the Kellgren-Lawrence grades, and the presence of osteophytes using the Osteoarthritis Research Society International grading criteria [20], and electronically measured hip joint space width at the supero-medial, apical and supero-lateral regions.

Inclusion and exclusion criteria

Participants with hip pain in the last 3 months, HHS ≤ 95 points, Kellgren-Lawrence grade 2 or 3 and/or joint space width ≤ 3 mm in one or both hips were defined as having hip OA (*n* = 27). Individuals with Kellgren-Lawrence grade 4 and joint space width < 1 mm or any major lower limb musculoskeletal or neurological conditions besides hip OA were excluded.

Data for the affected unilateral or most affected bilateral limb of individuals with hip OA were used for statistical analysis [12]. The most affected limb in those with bilateral hip OA was determined by the least joint space width. Ethical approval was granted by Griffith University Human Research Ethics Committee (GU Ref No: PES/23/08/HREC), Queensland Health, Health and Medical Research Human Research Ethics Committee (HREC/13/QPAH/207), and Monash University Human Research Ethics Committee (10754), and all participants provided written informed consent prior to commencement of the study.

### Anthropometric measures

Height was measured using a stadiometer with the removal of footwear. Weight was measured via an electronic scale with the removal of footwear and heavy clothing. Body mass index (BMI) was calculated.

### Hip pain and function

Hip pain and function were evaluated using the validated Hip Disability and Osteoarthritis Outcome Score (HOOS) [21]. The HOOS is composed of 40 items and assesses patient-relevant outcomes in five subscales: pain (10 items), symptoms (5 items), activity of daily living (17 items), sport and recreation function (4 items), and hip-related quality of life (4 items). Each question is scored from 0 to 4 (5 Likert boxes). For each subscale, the scores are summarized and transformed into a worst to best scale ranging from 0 to 100 scale. Higher scores refer to better outcome: 0 representing extreme problems and 100 representing no problems.

### Hip muscle CSA and fat infiltration

**Fig. 1:**
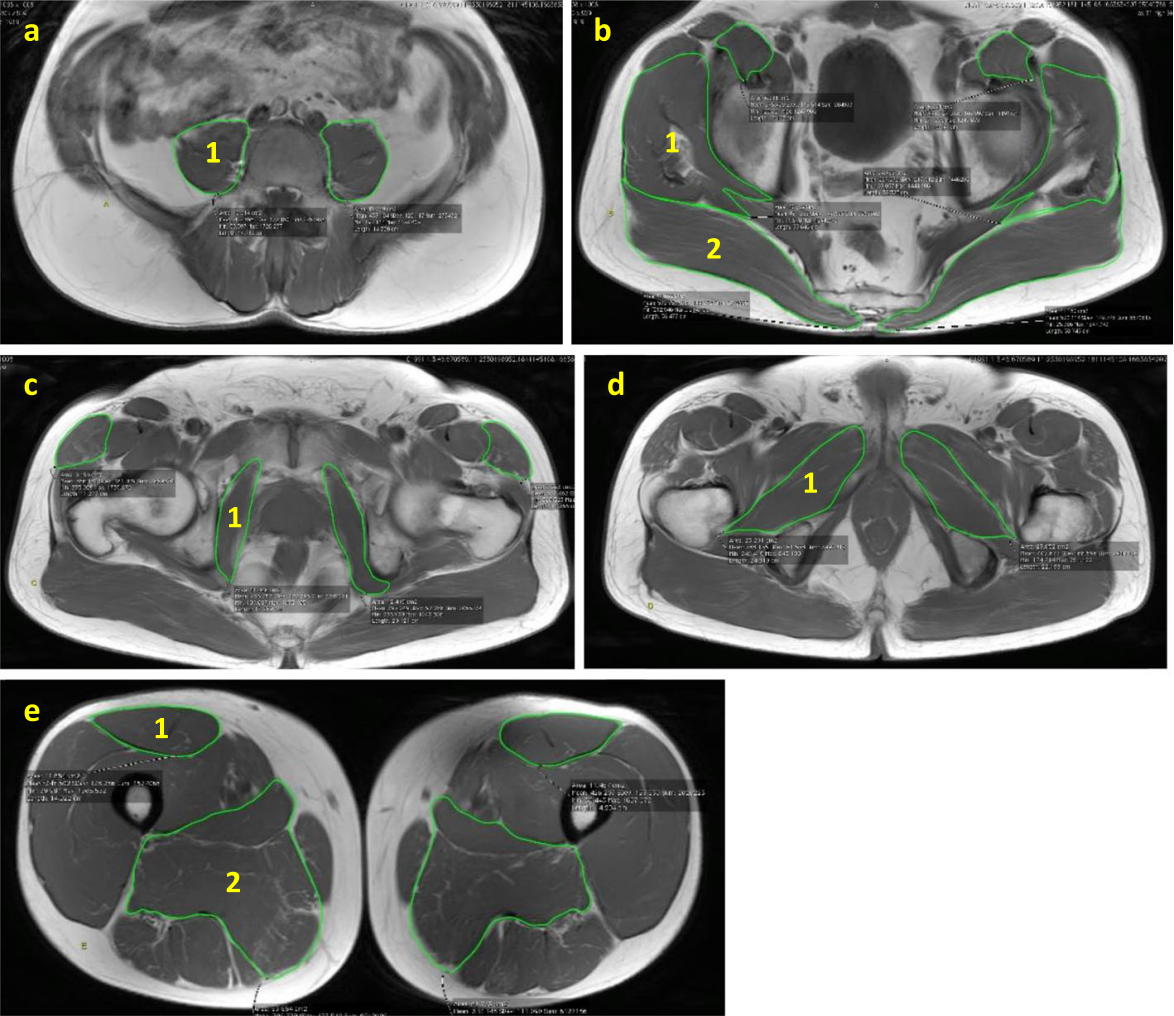
Cross-sectional area measurements of (**a**) 1. Psoas major; (**b**) 1. Gluteus medius and gluteus minimus, 2. Gluteus maximus; (**c**) 1. Obturator internus; (**d**) 1. Obturator externus; (**e**) 1. Rectus femoris, 2. Adductor longus and adductor magnus

Participants underwent magnetic resonance imaging (MRI) of the pelvis and leg (starting from above iliac crest down to knee, bilateral) using a 3.0 T MRI unit (Phillips Healthcare Ingenia). Participants were positioned in supine position with body coil arrays superiorly placed on lower limbs and legs in 15° of hip internal rotation, secured together with a strap. Hip muscle CSA was measured on axial images obtained using a T1 weighted 2-dimensional fast spin echo sequence (repetition time 731.6 msec, echo time 6.5 msec, flip angle 90°, slice thickness 10 mm, pixel matrix 0.47 mm × 0.47 mm, and 960 × 960 matrix). The CSA of hip muscles was measured from five regions (Fig. 1), adapted from a previous study [22]: (a) Iliac crest: psoas major; (b) Upper border of the acetabulum: gluteus maximus, gluteus medius and gluteus minimus; (c) Lower border of the acetabulum: obturator internus; (d) Ischial tuberosity: obturator externus; (e) Just below the gluteus maximus muscle: adductor longus, adductor magnus, and rectus femoris. Muscle CSA was measured by tracing the border of each muscle using the software Osirix (University Hospital of Geneva, Geneva, Switzerland) on an independent workstation. A reader, trained by a radiologist, who was blinded to the participant characteristics, hip pain and function, measured the hip muscle CSA twice with 1 week interval, and the average was taken as the muscle CSA. The intra-observer reproducibility (intra-class correlation coefficient) ranged from 0.78 to 1.00. Fat infiltration of hip muscles was measured on axial images and categorised into grade 0: no fat infiltration, grade 1: 1–10% fat infiltration, grade 2: 11–50% fat infiltration, and grade 3: > 50% fat infiltration. The intra-observer reproducibility (intra-class correlation coefficient) of our measurement was 0.99.

### Statistical analysis

Participant characteristics were tabulated. Multiple linear regression was used to examine the associations of hip muscle CSA with hip pain and function (HOOS scores) of the target hip, adjusted for age and gender. A *p*-value of less than 0.05 (two-tailed) was considered statistically significant. All statistical analyses were performed using the IBM Statistical Package for the Social Sciences (SPSS, Chicago, IL) software, version 24.

## Results

The characteristics of participants are shown in Table 1. The mean age of the participants was 63.2 years with a mean BMI of 28.0 kg/m^2^ and 66.7% being female. The mean joint space width of the study hip was 2.39 mm, and the majority (92.6%) of the participants had mild-to-moderate hip OA (i.e. Kellgren-Lawrence grade ≤ 3).

**Table 1:** Characteristics of study participants

	*N* = 27
Age, years	63.2 (7.6)
Female, n (%)	18 (66.7)
Body mass index, kg/m^2^	28.0 (4.1)
Joint space width, mm	2.39 (0.98)
Kellgren-Lawrence grade, n (%)	
1	1 (3.7)
2	11 (40.7)
3	13 (48.1)
4	2 (7.4)
**Hip muscle cross-sectional area, cm**^**2**^	
***Flexors***	
Psoas major	13.9 (3.7)
Rectus femoris	8.0 (2.3)
Flexors total^a^	21.8 (5.7)
***Extensors***	
Gluteus maximus	46.1 (10.2)
***Adductors***	
Adductor longus and magnus	40.3 (10.8)
***Abductors***	
Gluteus medius and minimus	32.4 (6.9)
***Rotators***	
Obturator internus	11.9 (2.0)
Obturator externus	26.5 (4.7)
Rotators total^b^	38.5 (5.7)
**HOOS scores**	
Pain	66.5 (21.6)
Symptoms	66.7 (18.6)
Activity of daily living	72.6 (24.2)
Sport and recreation function	60.7 (28.7)
Hip-related quality of life	52.3 (25.0)

Data presented as mean (standard deviation) or n (%).

*HOOS* Hip Disability and Osteoarthritis Outcome Score

^a^Psoas major + Rectus femoris; ^b^Obturator internus + Obturator externus

The associations between hip muscle CSA and HOOS scores are presented in Table 2. Although the CSA of psoas major, flexors total, adductor longus and magnus was positively associated with HOOS pain score in univariable analyses, there were no significant association between the CSA of hip muscles and HOOS pain score in multivariable analyses adjusted for age and gender. Greater CSA of adductor longus and magnus was associated with better hip-related quality of life in multivariable analyses (regression coefficient 1.4, 95% confidence interval (CI) 0.2 to 2.7, *p* = 0.02). There was a trend towards an association between greater CSA of psoas major (regression coefficient 3.6, 95% CI − 0.5 to 7.7, *p* = 0.08) and flexors total (psoas major and rectus femoris; regression coefficient 2.3, 95% CI − 0.3 to 5.0, *p* = 0.08) and better hip-related quality of life, achieving borderline significance. The CSA of other hip muscles was not significantly associated with hip-related quality of life.

**Table 2:** Relationship between hip muscle cross-sectional area and HOOS outcomes

	Univariable Regression coefficient (95% CI)	*P*-value	Multivariable Regression coefficient (95% CI)*	*P*-value*
Pain				
*Flexors*				
Psoas major	2.8 (0.6, 4.9)	0.01	2.8 (−0.9, 6.4)	0.13
Rectus femoris	3.4 (−0.1, 7.0)	0.06	2.4 (−2.9, 7.7)	0.36
Flexors total	1.7 (0.3, 3.1)	0.02	1.7 (−0.7, 4.1)	0.16
*Extensors*				
Gluteus maximus	0.5 (−0.3, 1.4)	0.20	−0.03 (−1.4, 1.3)	0.97
*Adductors*				
Adductor longus and magnus	0.9 (0.1, 1.6)	0.03	0.9 (−0.2, 2.1)	0.10
*Abductors*				
Gluteus medius and minimus	0.4 (−0.8, 1.7)	0.48	−0.7 (−2.6, 1.2)	0.45
*Rotators*				
Obturator internus	−0.9 (−5.4, 3.7)	0.70	−0.4 (− 5.4, 4.5)	0.86
Obturator externus	0.5 (−1.6, 2.5)	0.65	−1.3 (−3.9, 1.3)	0.30
Rotators total	0.2 (−1.5, 1.9)	0.84	−0.8 (− 2.7, 1.2)	0.41
Hip-related quality of life				
*Flexors*				
Psoas major	3.3 (0.9, 5.7)	0.01	3.6 (− 0.5, 7.7)	0.08
Rectus femoris	4.4 (0.3, 8.4)	0.04	3.8 (−2.2, 9.8)	0.20
Flexors total	2.1 (0.5, 3.7)	0.01	2.3 (−0.3, 5.0)	0.08
*Extensors*				
Gluteus maximus	0.9 (−0.1, 1.8)	0.07	0.6 (− 0.9, 2.2)	0.39
*Adductors*				
Adductor longus and magnus	1.1 (0.3, 2.0)	0.01	1.4 (0.2, 2.7)	**0.02**
*Abductors*				
Gluteus medius and minimus	0.6 (−0.9, 2.0)	0.44	−0.6 (−2.8, 1.6)	0.57
*Rotators*				
Obturator internus	0.2 (−5.1, 5.4)	0.95	1.2 (−4.4, 6.9)	0.66
Obturator externus	0.8 (−1.5, 3.1)	0.48	−1.1 (−4.0, 1.8)	0.44
Rotators total	0.5 (−1.4, 2.4)	0.61	−0.5 (−2.6, 1.7)	0.66

*Adjusted for age and gender

*HOOS* Hip Disability and Osteoarthritis Outcome Score, *CI* confidence interval

Greater CSA of adductor longus and magnus was associated with better function in activity of daily living (regression coefficient 1.3, 95% CI 0.1 to 2.6, *p* = 0.04) and in sport and recreation (regression coefficient 1.6, 95% CI 0.1 to 3.0, *p* = 0.04). The CSA of hip muscles was not significantly associated with HOOS symptom score in multivariable analyses (Supplementary Table 1).

There was low variation in the levels of fat infiltration of hip muscles, with fat infiltration of 1–10% or 11–50% (Supplementary Table 2). None of the fat infiltration variables were associated with hip pain and function outcomes in univariable linear regression analyses (all *p* > 0.20). Including fat infiltration in the regression models did not change the results (data not shown).

## Discussion

To our knowledge, this is the first study to examine the relationship between hip muscle CSA and hip pain and functional outcomes in community-based individuals with mild-to-moderate hip OA. There was a positive association between greater CSA of hip adductors and better function (activity of daily living and sport and recreation) and quality of life. There was also a non-significant trend towards an association between greater CSA of hip flexors and better quality of life. These findings suggest that targeting the hip adductor musculature in treatment may lead to improved hip function and quality of life among individuals with mild-to-moderate hip OA.

We found that greater CSA of hip adductors was associated with better functional outcomes in those with mild-to-moderate hip OA. The most significant and consistent associations were found for the hip adductors, including adductor longus and magnus. The hip adductor muscles play an important role in balancing the pelvis during standing and walking and for overall hip stability and injury prevention. Previous studies reported lower volume and CSA of hip adductor muscles and decreased hip adductor strength in people with hip OA compared with healthy controls [10, 22]. Other studies investigating pain and injury reported a lower hip adduction/abduction strength ratio in soccer players with groin pain compared with those without groin pain [15] and that hip adductor strength was decreased both preceding and during the onset of groin pain in football players [16]. Although these studies investigated adductor strength rather than CSA, the findings indicate the importance of hip adductor muscles when considering pain and recovery for better function.

There was a trend that larger CSA of hip flexors would be associated with better quality of life in our study. Hip flexors are used for a variety of everyday functional activities such as advancing the lower extremity during gait, running, or lifting the leg when going up steps. Although decreased hip flexor strength has been reported in people with hip OA compared with healthy controls [10, 22], no previous studies have examined the relationship between hip flexors and hip pain or functional outcomes. While gluteal muscle atrophy (i.e. decreased muscle volume) was associated with the presence and clinical severity of hip OA [23–26], one study found a weak negative correlation between gluteus medius muscle fiber CSA and hip pain [17]. In contrast, our study did not find an association between CSA of gluteus medius and minimus muscle and hip pain or function. The gluteus medius and gluteus minimus muscles were measured together in our study, as the border between the two muscles was indistinguishable on our scans; as a result, the association between gluteus medius and hip pain or function may have been overlooked.

There is evidence that larger CSA of hip muscles may result in greater muscle strength [7, 8, 22] which would allow efficient force distribution within the joint and improvement of hip stability, resulting in better function. It is also plausible that better function facilitates more use and therefore larger CSA of the hip muscles. Our results suggest that there are potential muscles that could be targeted in those with hip OA to improve functional outcomes. This will need to be tested in clinical trials. Clinical guidelines for the treatment of hip OA recommend education, strength training and exercise programs [27–29]. A systematic review of 13 cross-sectional studies suggested the need to target muscle weakness in the clinical management of hip OA [9]. In persons with hip OA, the greatest reduction in muscle strength of the affected leg compared with the contralateral leg was seen for hip flexors and extensors, with less consistent data for hip adductors and abductors [9]. However, there were consistent data for lower hip abductor strength in people with hip OA compared with healthy controls [9, 10, 22–24, 30]. Adding to the literature, our study found beneficial associations of larger CSA of hip adductors with better function and quality of life in hip OA. Taken together, these data suggest that targeting these hip muscles may have significant implications for reducing the burden of hip OA in the community, where pain, disability and impaired quality of life are growing concerns in the current population.

This study had limitations. It is a cross-sectional study with a moderate sample size. Whether there is a temporal relationship between hip muscle CSA and functional outcomes could not be investigated. The moderate sample size limited the power of the study to detect an association between CSA of some hip muscles and pain and functional outcomes. The CSA of hip muscles was not significantly different between the right and left hips in the 11 participants with bilateral hip OA (data not shown). The most affected hip in participants with bilateral hip OA was the study hip for statistical analyses. The results did not change when the average CSA of right and left hip muscles were examined in those with bilateral hip OA (data not shown). Due to the lack of demarcation between muscles, some specific muscles could not be segmented individually and instead were grouped for CSA measurement, such as the adductors, and gluteus medius and gluteus minimus muscles. Identifying specific muscles could shed further light on which muscles contribute to better hip outcomes. Consistency with regards to anatomical positions for muscle CSA measurement was another issue. The slice thickness may have surpassed some regions due to the difference in terms of where the initial slice began or the difference in body size among participants. As shown in a systematic review, segmentation of CSA on a single slice increased volume errors [31]. These types of measurement errors were likely to be at random and have resulted in underestimation of the magnitude of observed associations. Furthermore, studies looking at muscle volume and adiposity are emerging areas that may provide additional information and overcome some of the limitations of examining CSA alone. We were only able to adjust for limited numbers of confounders in the statistical analyses, and we have controlled for the difference in body size by adjustment for gender. The strengths of this study include the high reproducibility of the MRI measurement of hip muscle CSA. A full-length scan from the iliac crest to the knee allowed the CSA measurement of hip muscles of different functional groups. Furthermore, there is evidence for the validity of CSA measurement of hip muscles against muscle strength and severity of hip OA [22]. HOOS is validated and well accepted in the OA scientific literature and clinical settings [21]. It is easy to use within clinical practice to follow patients with hip OA over time and is suitable to use in research as a disease-specific questionnaire [21].

### Conclusions

This study found that greater CSA of hip adductors was associated with better function and quality of life in individuals with mild-to-moderate hip OA. Furthermore, a similar trending association was found between hip flexor CSA and quality of life. These findings, while need to be tested in clinical trials, suggest that targeting hip adductor and flexor muscles may have a beneficial effect on improving function and quality of life in those with mild-to-moderate hip OA.

## Supplementary information

**Additional file 1: Table S1.** Relationship between hip muscle cross-sectional area and HOOS outcomes. **Table S2.** Fat infiltration of hip muscles.

## Data Availability

The datasets used and/or analysed during the current study are available from the corresponding author on reasonable request.

## Abbreviations

CSA: Cross-sectional area
OA: Osteoarthritis
HOOS: Hip Disability and Osteoarthritis Outcome Score
BMI: Body mass index
CI: Confidence interval
