# Supplementary material for: Association between hip muscle cross-sectional area and hip pain and function in individuals with mild-to-moderate hip osteoarthritis: a cross-sectional study
Source: BMC Musculoskelet Disord. 2020 May 21;21:316. doi: 10.1186/s12891-020-03348-5 (PMC7240926; doi:10.1186/s12891-020-03348-5)
Supplement: Supplementary file 1 — Additional file 1: Table S1. Relationship between hip muscle cross-sectional area and HOOS outcomes. Table S2. Fat infiltration of hip muscles. [file 12891_2020_3348_MOESM1_ESM.docx]

**Supplementary Table 1. Relationship between hip muscle cross-sectional area and HOOS outcomes**

|  | Univariable  Regression coefficient (95% CI) | P-value | Multivariable  Regression coefficient (95% CI)* | P-value* |
| --- | --- | --- | --- | --- |
| Symptoms |  |  |  |  |
| *Flexors* |  |  |  |  |
| Psoas major | 2.3 (0.4, 4.1) | 0.02 | 1.7 (-1.5, 4.8) | 0.28 |
| Rectus femoris | 2.9 (-0.2, 6.0) | 0.07 | 1.4 (-3.1, 5.9) | 0.53 |
| Flexors total | 1.4 (0.2, 2.6) | 0.02 | 1.0 (-1.0, 3.1) | 0.32 |
| *Extensors* |  |  |  |  |
| Gluteus maximus | 0.4 (-0.4, 1.1) | 0.34 | -0.5 (-1.6, 0.7) | 0.40 |
| *Adductors* |  |  |  |  |
| Adductor longus and magnus | 0.6 (-0.03, 1.3) | 0.06 | 0.5 (-0.5, 1.5) | 0.34 |
| *Abductors* |  |  |  |  |
| Gluteus medius and minimus | 0.5 (-0.6, 1.6) | 0.35 | -0.5 (-2.1, 1.1) | 0.54 |
| *Rotators* |  |  |  |  |
| Obturator internus | 0.2 (-3.7, 4.0) | 0.94 | 0.7 (-3.4, 4.9) | 0.72 |
| Obturator externus | 0.8 (-0.9, 2.5) | 0.34 | -0.6 (-2.8, 1.6) | 0.58 |
| Rotators total | 0.6 (-0.8, 2.0) | 0.37 | -0.1 (-1.8, 1.5) | 0.87 |
| Activity of daily living | |  |  |  |
| *Flexors* |  |  |  |  |
| Psoas major | 2.6 (0.1, 5.1) | 0.04 | 3.1 (-1.1, 7.3) | 0.14 |
| Rectus femoris | 3.6 (-0.5, 7.6) | 0.08 | 3.8 (-2.2, 9.8) | 0.21 |
| Flexors total | 1.7 (0.1, 3.3) | 0.04 | 2.1 (-0.6, 4.9) | 0.12 |
| *Extensors* |  |  |  |  |
| Gluteus maximus | 0.4 (-0.5, 1.4) | 0.36 | 0.02 (-1.5, 1.6) | 0.98 |
| *Adductors* |  |  |  |  |
| Adductor longus and magnus | 0.9 (0.1, 1.8) | 0.04 | 1.3 (0.1, 2.6) | **0.04** |
| *Abductors* |  |  |  |  |
| Gluteus medius and minimus | 0.3 (-1.2, 1.7) | 0.70 | -0.7 (-2.9, 1.6) | 0.54 |
| *Rotators* |  |  |  |  |
| Obturator internus | -0.7 (-5.7, 4.4) | 0.79 | 0.2 (-5.5, 6.0) | 0.94 |
| Obturator externus | 0.1 (-2.2, 2.4) | 0.94 | -1.6 (-4.7, 1.4) | 0.27 |
| Rotators total | -0.03 (-1.9, 1.9) | 0.97 | -0.8 (-3.1, 1.4) | 0.44 |
| Sport and recreation function | |  |  |  |
| *Flexors* |  |  |  |  |
| Psoas major | 3.6 (0.7, 6.4) | 0.02 | 3.7 (-1.2, 8.5) | 0.13 |
| Rectus femoris | 5.1 (0.4, 9.7) | 0.04 | 4.6 (-2.4, 11.5) | 0.19 |
| Flexors total | 2.3 (0.5, 4.1) | 0.02 | 2.5 (-0.6, 5.7) | 0.11 |
| *Extensors* |  |  |  |  |
| Gluteus maximus | 0.5 (-0.6, 1.6) | 0.38 | -0.5 (-2.3, 1.3) | 0.54 |
| *Adductors* |  |  |  |  |
| Adductor longus and magnus | 1.3 (0.3, 2.2) | 0.01 | 1.6 (0.1, 3.0) | **0.04** |
| *Abductors* |  |  |  |  |
| Gluteus medius and minimus | 0.7 (-1.0, 2.4) | 0.41 | -0.6 (-3.2, 2.0) | 0.64 |
| *Rotators* |  |  |  |  |
| Obturator internus | -0.9 (-6.9, 5.1) | 0.77 | -0.3 (-7.0, 6.3) | 0.93 |
| Obturator externus | 0.9 (-1.8, 3.6) | 0.51 | -1.4 (-4.9, 2.0) | 0.40 |
| Rotators total | 0.5 (-1.7, 2.7) | 0.65 | -0.7 (-3.3, 1.8) | 0.55 |

*Adjusted for age and gender

HOOS: Hip Disability and Osteoarthritis Outcome Score; CI: confidence interval

**Supplementary Table 2. Fat infiltration of hip muscles**

|  | Fat infiltration 1-10% | Fat infiltration 11-50% |
| --- | --- | --- |
| Psoas major | 29.6% | 70.4% |
| Rectus femoris | 100% | 0 |
| Gluteus maximus | 88.9% | 11.1% |
| Adductor longus and magnus | 92.6% | 7.4% |
| Gluteus medius and minimus | 7.4% | 92.6% |
| Obturator internus | 44.4% | 55.6% |
| Obturator externus | 20.0% | 80.0% |
